# Essential role of MHC II in the antitubercular efficacy of pyrazinamide

**DOI:** 10.1128/aac.01264-25

**Published:** 2025-12-19

**Authors:** Elise A. Lamont, Shannon L. Kordus, Michael D. Howe, Ziyi Jia, Nathan Schacht, Muzafar Ahmad Rather, Gebremichal Gebretsadik, Anthony D. Baughn

**Affiliations:** 1 Department of Microbiology and Immunology, University of Minnesota 5635, Minneapolis, Minnesota, USA; City St George's, University of London, London, United Kingdom

**Keywords:** pyrazinamide, *Mycobacterium tuberculosis*, CD4 T cells, interferon-gamma, oxidative burst, MHC II

## Abstract

Antibacterial drug mechanisms have traditionally been examined through a drug-pathogen lens, with limited attention to host influences on drug activity. However, growing evidence suggests that the host environment is crucial for antibacterial efficacy. Pyrazinamide (PZA), a key component of modern tuberculosis therapy, exemplifies this complexity, exhibiting potent *in vivo* activity despite its inability to reduce *Mycobacterium tuberculosis* viability in standard *in vitro* culture. Here, using macrophage and murine infection models, we identify a critical role for CD4^+^ T cell-dependent cell-mediated immunity in PZA’s antitubercular action. Using MHC class II knockout mice, we demonstrate that CD4 T-cell help is essential for PZA efficacy. While interferon gamma (IFN-γ) is required for PZA-mediated clearance of *M. tuberculosis* at extrapulmonary sites, bacterial reduction in the lungs occurs, independent of IFN-γ signaling. We show that PZA leverages cell-mediated immunity in part through activation of the oxidative burst. Our findings underscore the need to incorporate host factors into antibacterial drug evaluation and highlight potential avenues for host-directed therapies and adjunctive antibiotics in first- and second-line tuberculosis treatment.

## INTRODUCTION

“…when pyrazinamide is used alone, we are never entirely certain what will happen,” Walsh McDermott, a pioneer of multi-drug chemotherapy for tuberculosis (TB), exclaimed in his 1956 paper promoting a pyrazinamide (PZA) and isoniazid (INH) combinatorial regimen (1). This uncertainty was widely recognized by McDermott’s contemporaries in the TB field and prompted speculation that the host contributed to variability in drug efficacy, particularly when treatment failure could not be attributed to drug resistance (1). These observations helped to define drug therapy as a tripartite interaction among the drug, parasite, and host (1, 2). In fact, PZA’s initial discovery as a sterilizing TB drug occurred through *in vivo* studies in *Mycobacterium tuberculosis* (*Mtb*)-infected mice (3, 4). Under standard *in vitro* broth and agar conditions, PZA displayed no discernible growth effects against *Mtb* cultures (5–7). Thus, the host environment was considered a fertile basis for uncovering drug mechanisms and activities, especially for drugs that may have disparate effects under *in vitro* and *in vivo* conditions (2).

Despite this historic insight, the mechanism of antimicrobial drug action has largely been untethered from the host response, partially due to the complexity and reproducibility of pharmacodynamic studies in animal models and the patient care setting (8, 9). In recent years, however, select antibiotics were shown to influence immune processes, such as antibiotic-induced neutrophil extracellular traps or altering bacterial metabolism to increase reactive oxygen species (ROS) (10–12). These examples demonstrate antibiotics acting on host responses, but the reverse—the host influencing antibiotic efficacy—remains less explored. Consequently, drug discovery and optimization have relied on reductionist approaches centered on the drug’s minimum inhibitory concentration in standardized bacteriologic media (13). Although growth media can be supplemented with purified antimicrobial peptides and small chemicals to mimic aspects of the host, these systems cannot recapitulate the full complexity of the host environment or its effects on drug activity and localization. This limitation is exemplified by PZA, the only clinically used TB drug with an unresolved mechanism of action (5, 14–16). While multiple models have been proposed to explain PZA’s mechanism, only the PZA protonophore model incorporates host contribution in PZA activity (14, 17, 18). Although this model has been challenged, acidic pH as it relates to the intracellular host environment remains a defining feature of host-mediated potentiation of PZA and a critical component of PZA’s activity *in vivo* (19, 20).

Recent evidence from both animal models and human granuloma specimens underscores the importance of the host environment in determining PZA penetration, availability, and activity (21–28). Several studies show that *Mtb* susceptibility to PZA varies, depending on the location of the bacilli within granulomas and host cells (23, 25, 29). In cell models of TB infection, PZA accumulates within acidified phagosomes, where it exhibits maximum efficacy (20, 29). Consistently, PZA efficacy is reduced or abolished in mouse strains with impaired T cell-dependent immunity (21, 23–26). Also, PZA fails to sterilize extracellular *Mtb* bacilli residing in the near-neutral pH caseum of the C3HeB/FeJ mouse strain (25). In contrast, PZA retained modest cidal activity against *Mtb* when added to acidic caseum purified from a rabbit model, indicating that the antimicrobial effect results from the low-pH microenvironment rather than the intrinsic properties of the caseum itself (30, 31). Although PZA-mediated killing of extracellular *Mtb* can be achieved in caseum at an acidic pH, PZA is likely most potent against *Mtb* localized to intracellular environments, like the phagosome.

Within this intracellular environment, several host defense pathways are engaged against *Mtb*, some of which are linked to PZA potentiation. Additional host-derived stressors that enhance or bioactivate PZA likely remain to be identified. Defining these host responses will clarify the mechanisms underlying host-dependent PZA activity and inform development of host-directed therapies to augment PZA activity, shorten treatment duration, and improve outcomes for patient populations at risk of drug failure.

We have previously shown that sublethal PZA exposure sensitizes *Mtb* to ROS, enhancing bactericidal activity (32). Host-derived ROS generated via interferon gamma (IFN-γ)-induced NADPH oxidase activation constitutes a key macrophage defense strategy to restrict *Mtb* growth (33–35). We have also shown that IFN-γ activation of macrophages increases PZA-mediated killing of *Mtb* (32). These data suggest that ROS is another exciting host factor that contributes to PZA’s conditional efficacy *in vivo*. In this study, we further investigate the role of ROS, the oxidative burst, and the CD4^+^ T-cell immune response in PZA potentiation. Using complementary cell and animal models of TB infection, we show that the oxidative burst and MHC class II (MHC II) expression are essential for the potent bactericidal activity of PZA *in vivo*. This previously unknown mechanism provides a rationale for host-directed strategies aimed at amplifying immune responses to enhance PZA activity.

## RESULTS

**Fig 1:**
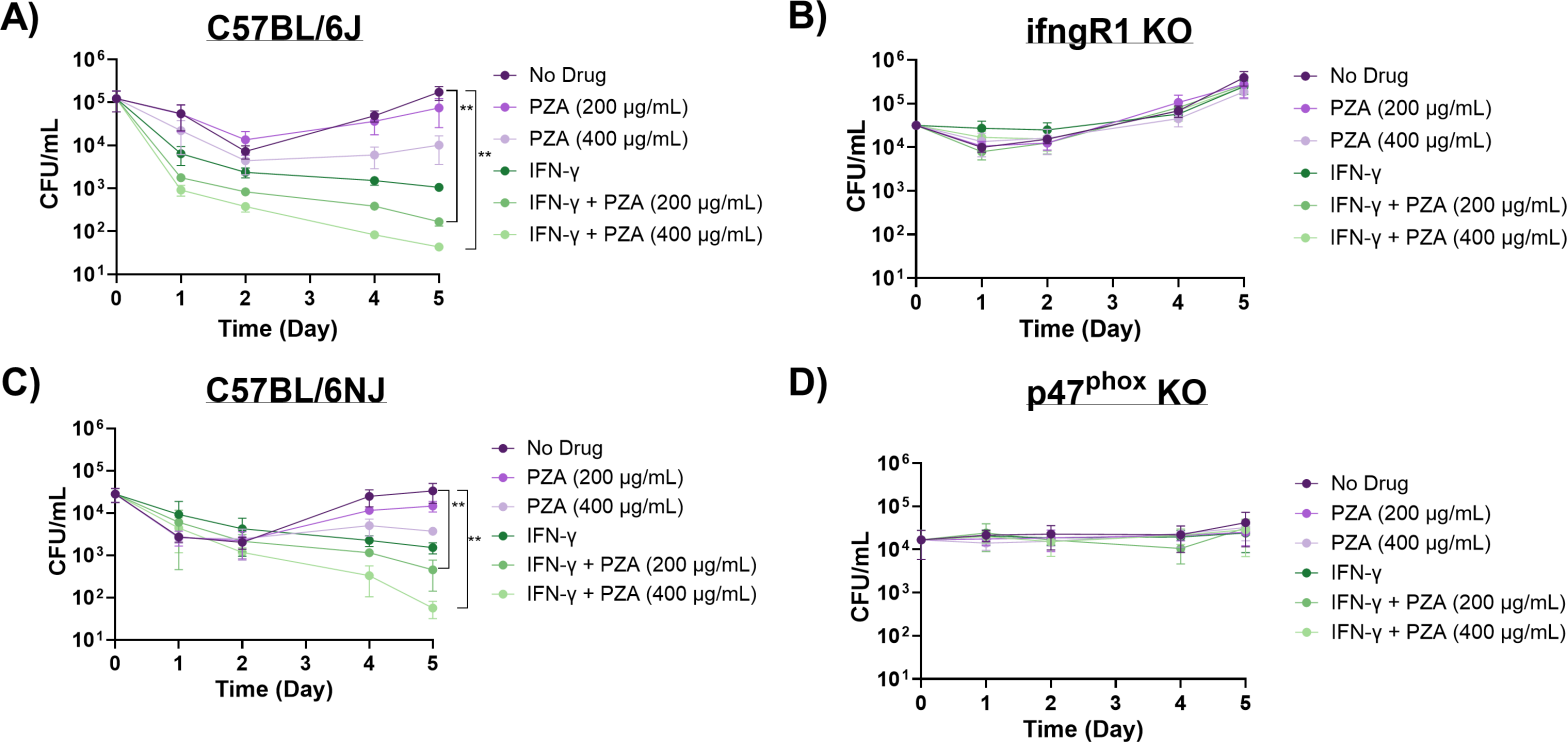
PZA activity is dependent on the oxidative burst. PZA bactericidal activity in *Mtb* strain H37Rv infected (**A**) C57BL/6J, (**B**) *ifngR1* KO, (**C**) C57BL/6NJ, and (**D**) *p47^phox^* KO BMDMs. Infected resting and IFN-γ activated BMDMs were untreated or treated with PZA (200 µg/mL or 400 µg/mL). Cultures were plated for bacterial enumeration at indicated timepoints. Statistical significance was calculated based on multiple comparisons with two-way ANOVA with Bonferroni correction. **P < 0.005*.*

We selected bone marrow-derived macrophages (BMDMs) from well-established genetic knockout (KO) mouse strains gamma-interferon receptor 1 (*ifngR1*; deficient in gamma-interferon signaling) KO and *phox47* KO (deficient in producing p47phox) and wild-type (WT) counterparts (C57BL/6J and C57BL/6NJ) in *Mtb* infection assays to further explore the role of the oxidative burst in PZA activity. To promote the oxidative burst, we activated BMDMs with IFN-γ prior to PZA treatment. IFN-γ upregulates NADPH oxidase, a multi-subunit enzyme complex that catalyzes molecular oxygen to generate superoxide anions, which are later converted to multiple ROS within the phagosome (35, 36). Bacterial burden was assessed throughout the 5-day post-infection (p.i.) period. Similar to our previous findings in *Mtb*-infected RAW 264.7 and THP-1 macrophage-like cell lines, IFN-γ-activated BMDMs enhanced PZA-mediated killing of *Mtb* in both C57BL/6J and C57BL/6NJ WT BMDMs (32). The addition of PZA (200 µg/mL) to IFN-γ-treated macrophages resulted in a 2.65- and 1.51-log_10_ reduction in bacterial burden in C57BL/6J and C57BL/6NJ BMDMs, respectively, greater than PZA (200 µg/mL) alone at 5 days p.i. (Fig. 1A and C; Tables S1 and S2). Increased bacterial killing was also observed at days 4 and 5 p.i. in the IFN-γ and PZA (400 µg/mL) treatment compared to PZA (400 µg/mL) alone in BMDMs from both WT strains of mice (Fig. 1A and C; Tables S1 and S2). This elevated bactericidal activity is due to the synergy between IFN-γ and PZA as this combinatorial treatment resulted in a 0.5–1.4 greater log_10_ reduction than IFN-γ alone at day 5 p.i. for both WT strains (Fig. 1A and C; Tables S1 and S2).

IFN-γ and PZA synergy was abrogated in *ifngR1* KO BMDMs and was indistinguishable from the no-drug control (Fig. 1B). IFN-γ was unable to bind to IFNGR1, therefore preventing further signaling, including increasing the oxidative burst capacity. *phox47* KO BMDMs were subsequently used to directly assess the oxidative burst in PZA potentiation. p47^Phox^ is a critical regulatory subunit in the NADPH oxidase complex, which aids in ROS production (37). A previous study demonstrated that *p47^phox^*^−/−^ mice failed to control *Mtb* replication during early infection and that this loss of control was most likely attributed to diminished ROS production (38). Our results show that the addition of IFN-γ is unable to enhance the oxidative burst in BMDMs from *phox47* KO mice and consequently fails to alter PZA activity against *Mtb* (Fig. 1D). Together, these cell infection data suggest that the oxidative burst is an important host component for PZA-mediated killing of *Mtb*.

**Fig 2:**
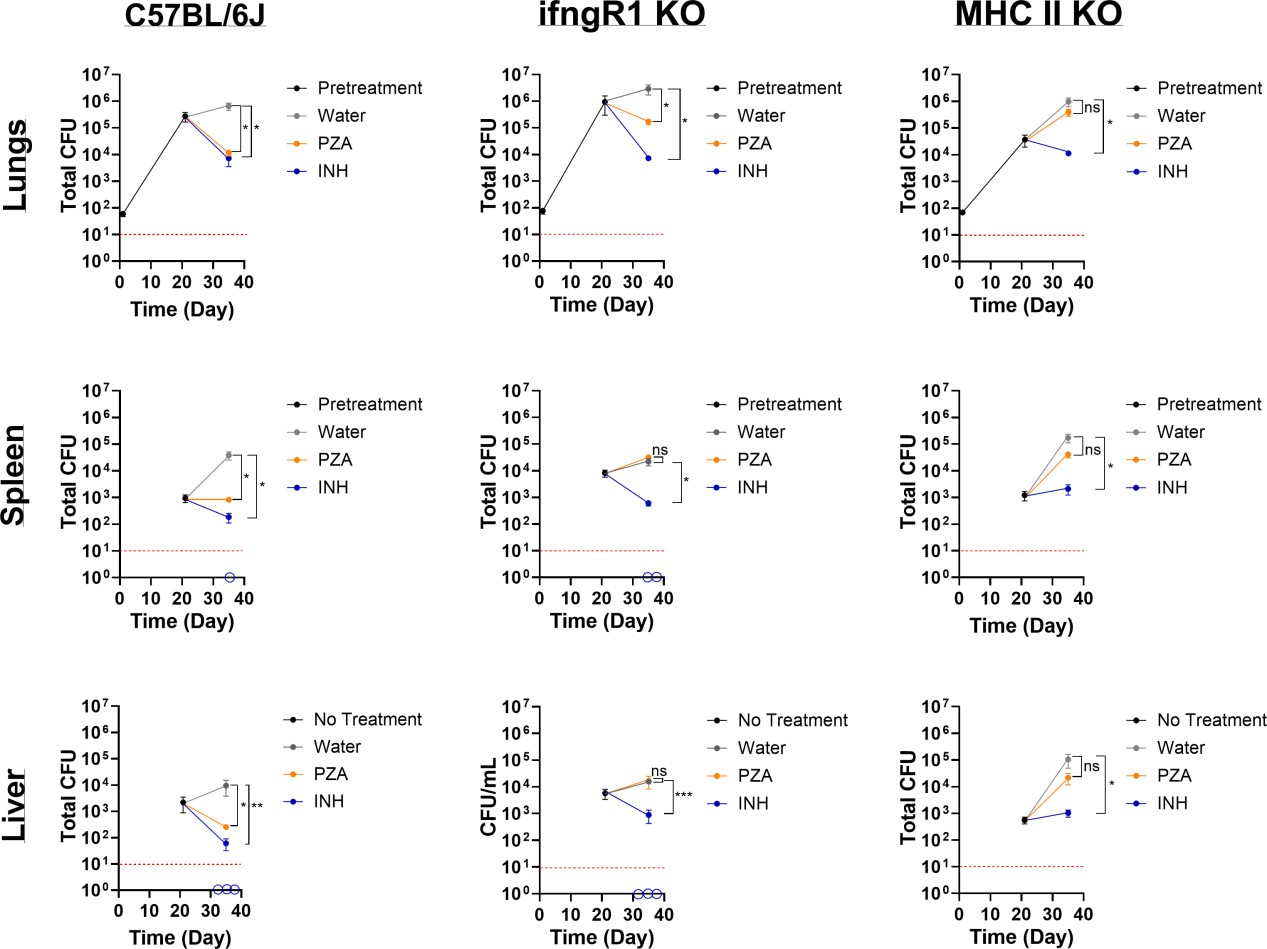
PZA failure in *ifngR1* KO and MHC II KO mice. *Mtb* strain H37Rv total bacterial burden in lungs, livers, and spleens of C57BL/6J, *ifngR1 KO*, and MHC II KO mice. All mice were infected with low-dose aerosol *Mtb*, and the infection was allowed to progress for 3 weeks in order for cell-mediated immunity to initiate (pretreatment). Mice were subsequently treated daily for 5 days a week for 2 weeks with either vehicle control (sterile water), PZA (150 mg/kg), and INH (30 mg/kg). Upon euthanasia, organs were removed, homogenized, and plated for bacterial enumeration at indicated timepoints. Empty circles on the *x*-axis indicate animals for which no bacterial growth was detected. Statistical significance was calculated based on paired two-tailed *t*-tests, and *P* values were adjusted for multiple comparisons.*P < 0.05, **P < 0.05, ***P < 0.0005. ns, not significant.

Based on previous studies examining PZA activity in immune-impaired mice and our own results highlighting the contribution of IFN-γ and the oxidative burst in PZA activity, we asked what the role of IFN-γ in PZA efficacy is *in vivo* (21, 23–26). *Mtb*-infected C57BL/6J (WT) and *ifngR1* KO mice were treated with controls (vehicle control [sterile water] or INH) or PZA 3 weeks p.i., commensurate with the initiation of cell-mediated immunity. Treatments were administered via daily oral gavage for 2 weeks, and lung, spleen, and liver bacterial burdens were assessed. As expected, PZA treatment in WT mice caused a reduction in bacterial load compared to vehicle control for all organs (Fig. 2). PZA treatment reduced *Mtb* bacterial load by 2.0, 1.5, and 1.5 log_10_ in lungs, spleens, and livers, respectively, compared to the vehicle control (Fig. 2). However, PZA treatment in *ifngR1* KO mice displayed disparate responses in the lungs versus extrapulmonary sites (Fig. 2). In the lungs of *ifngR1* KO mice, PZA-mediated killing of *Mtb* remained intact, albeit to a lesser extent (~1.2-log_10_ reduction in bacterial burden compared to 2.0-log_10_ reduction in WT counterparts; Fig. 2). However, in both *ifngR1* KO spleens and livers, PZA treatment was indiscernible from the vehicle control (Fig. 2). Drug failure in the *ifngR1* KO mice spleens and livers is specific to PZA as the control drug, INH, results in 2.5- and 1.0-log_10_ reductions in *Mtb* bacterial loads in spleens and livers, respectively (Fig. 2). In fact, some *ifngR1* KO mice treated with INH had no discernible bacterial growth in the spleens and livers (below limit of detection, Fig. 2).

IFN-γ-independent mechanisms exist within the lung and serve as significant contributors in the host response to pulmonary TB. For instance, CD4 T cells expressing CD153 (a tumor-necrosis factor [TNF] superfamily member) have been correlated with reduced lung *Mtb* load in non-human primates and humans independent of IFN-γ production (39). Furthermore, CD153 was shown to be critical in the control of *Mtb* replication in the lungs of infected mice (40). It should also be noted that CD153 is a ligand of CD30, a co-stimulatory receptor that was revealed to be necessary for CD4 T-cell control of *Mtb* expansion in the lung (41). Together, both receptor (CD30) and ligand (CD153) drive CD4 T-cell differentiation and multiple effector responses. Given the importance of CD4 T cells in pulmonary *Mtb* control combined with IFN-γ independence in some cell subsets, we proceeded to ask if CD4 T cells played a role in PZA activity in the lung. We selected MHC II KO mice to pursue this question as this mouse strain is deficient in MHC II and lacks CD4^+^ T cells (42). *Mtb* infection and drug treatment in MHC II KO mice were performed exactly as the previous *in vivo* experiment. Regardless of tissue type, PZA failed to make an appreciable difference in *Mtb* burden in MHC II KO mice (Fig. 2). Unlike in the *ifngR1* KO mice, PZA failed to reduce the number of *Mtb* bacilli from the pretreatment level (Fig. 2). While slight differences in *Mtb* load were noted for the spleen and liver (~0.4- and ~0.52-log_10_ decreases, respectively), these reductions were not statistically different from the vehicle control (Fig. 2). Treatment failure in MHC II KO mice appears to be restricted to PZA as INH reduced bacterial burden by 2 log_10_ in lungs and extrapulmonary organs (Fig. 2). These data suggest that the cell-mediated component of PZA potentiation is MHC II dependent.

## DISCUSSION

To conclude, this is the first study to specifically demonstrate MHC II expression, a strong inference for involvement of CD4 T cells as a major host factor responsible for the conditional efficacy of PZA *in vivo*. Both IFN-γ-dependent and IFN-γ-independent CD4 T cells are likely to play a key function in PZA activity that is specialized to host tissues. Based on our findings, IFN-γ^+^ CD4 T cells are anticipated to promote PZA action in extrapulmonary organs, while IFN-γ-independent CD4 T cells contribute to PZA-mediated *Mtb* clearance within the lung. These observations align with reports that IFN-γ-independent control of *Mtb* relies on CD4 T cell-derived GM-CSF and HIF-1α activation, whereas IFN-γ responses dominate in systemic tissues (40, 43). Together, these distinct arms of CD4 T-cell immunity may explain the variable PZA activity observed across lungs, spleens, and livers.

The discrepancy in PZA-mediated *Mtb* killing may partially be explained by recent findings demonstrating programmed cell death protein 1 (PD-1) repression of IFN-γ in the lungs to control host-induced immunopathology (40). In contrast, over 80% of bacterial replication is restricted by IFN-γ in the spleen (40). Thus, IFN-γ is likely to be a significant contributor to PZA potentiation at extrapulmonary sites where increased IFN-γ production is not detrimental to the host. Thus, IFN-γ is likely a major contributor to PZA potentiation at extrapulmonary sites where elevated IFN-γ responses are not detrimental to the host. These data support a model in which local immune regulation by PD-1 and other checkpoint molecules indirectly governs the extent of PZA activity by shaping the inflammatory milieu and balancing bacterial killing with tissue preservation.

While IFN-γ is critical to the oxidative burst, it also regulates pro- and anti-inflammatory cytokines, including TNF-α, IL-1, IL-6, IL-12, and IL-10 (44–46). It is possible that IFN-γ enhancement of PZA is due to the modification of these cytokines that are unrelated to the oxidative burst. For example, unique among the first-line TB drugs, PZA was shown to be active in highly inflamed lesions during early treatment and may explain why this drug is at its most potent in the first 2 months of therapy (27, 47). More recently, short-term blocking of IL-10 using anti-IL-10R1 antibody in *Mtb*-infected mice aided PZA-mediated bacterial clearance in 70% of treated mice (below the threshold of detection) compared to mice given PZA alone within 30 days post-treatment (48).

Beyond IFN-γ and cytokine effects, NADPH oxidase activity also appears to play an essential role in PZA efficacy. The phox47 subunit, which contributes to assembly and function of the NADPH oxidase complex, may influence PZA activation or distribution by several mechanisms. In addition to generating reactive oxygen species, NADPH oxidase alters endosomal pH, promotes lipid peroxidation, and modulates redox-sensitive enzymes that may enhance PZA bioactivation (49, 50). Because these mechanisms may not be unique to PZA, future studies should assess whether phox47 deficiency similarly affects the activity of other antitubercular drugs.

Future studies should further explore the contribution of specific subsets of CD4 T cells to PZA function. Particularly, CD30 KO and CD153 KO mice should be utilized to assess their contribution to PZA activity in the lungs. Elucidating CD30’s role in PZA-mediated *Mtb* clearance will also be essential to inform drug therapy decisions in TB-positive patients that also have relapsed or refractory lymphoma. Relapsed or refractory lymphoma patients may be prescribed the anti-CD30 monoclonal antibody brentuximab vedotin to target tumor cells; however, anti-CD30 drugs could diminish PZA activity in TB co-infected patients and consequently promote PZA resistance in *Mtb* (51, 52).

Importantly, this study also provides an explanation for Walsh McDermott’s perplexing observation that PZA monotherapy often elicited bewildering and unpredictable results (1). In particular, attention should be given to the host’s immune status regarding successful treatment with PZA. Host immune status, particularly CD4 T-cell competence and downstream effector pathways, such as MHC II, NADPH oxidase, and cytokine signaling, is likely a major determinant of PZA responsiveness. The sterilizing activity of PZA is likely conferred through its unique advantage against bacilli residing within the specialized niche of T cell-activated macrophages. Undoubtedly, understanding which patient populations may be unresponsive to PZA treatment will enable physicians to better tailor TB drug regimens to ensure successful *Mtb* sterilization and prevent future relapses.

These findings also highlight several opportunities for host-directed therapies and other antitubercular drugs to either enhance PZA activity or overcome deficiencies in the host response. Adjunctive therapies may include those that induce the autophagy pathway and thereby promote phagosome maturation and the oxidative burst. For example, a different antitubercular drug, bedaquiline, increases PZA efficacy by promoting PZA accumulation in phagosomes by inducing lysosome acidification via autophagy (20, 53). Commercially available host-directed drugs, such as rapamycin and metformin, that also induce autophagy may potentiate PZA and should be further evaluated for repurposing in the anti-TB drug regimen (54–58). Additionally, the development of recombinant forms of CD153 will allow for CD30 stimulation and promote IFN-γ-independent CD4 T cells and other effector functions that drive PZA activity in the lungs. In a broader context, our data demonstrate the need to include the host during the initial stages of drug development. It is possible that several promising drugs are overlooked and eliminated when solely using *in vitro* methods that focus on the drug-parasite paradigm. Thus, it is essential that we restore the drug-parasite-host relationship for the consideration and evaluation of newly discovered antitubercular drugs and anti-TB combinatorial therapies.

## MATERIALS AND METHODS

### Mice

WT C57BL/6J (#000664), WT C57BL/6NJ (#005304), B6N.129S2-*Ncf1^tm1Shl^*/J (PHOX47^phox−/−^, #027331), B6129S7-*IfngR1^tm1Agt^*/J (IFNGR1^−/−^, #003288), and B6.129S2-*H2^dlAb1-Ea^*/J (MHC II^−/−^, #003584) were purchased from the Jackson Laboratory. Mice were 4–7 weeks of age. All animals were housed in an ABSL-3 vivarium except for mice used for bone marrow extraction. Mice were provided with irradiated standard rodent chow and sterilized water *ad libitum*.

### Cell line and primary cells

CMG14-12 cells (C3H male) were cultured in Dulbecco’s Modified Eagle Medium/Nutrient Mixture F-12 (DMEM/F12) supplemented with 10% heat-inactivated fetal bovine serum (HI-FBS) and 1% penicillin/streptomycin mixture at 37°C in a 5% CO_2_ incubator (59). CMG14-12 conditioned medium was filtered through a 0.22 µm sterile syringe unit with a polyethersulfone membrane, aliquoted, and frozen at −70°C until further use. Macrophages were harvested separately from the bone marrow of female C57BL/6J, C57BL/6NJ, B6N.129S2-*Ncf1^tm1Shl^*/J, and B6129S7-*IfngR1^tm1Agt^*/J mice and maintained in DMEM/F12 supplemented with HI-FBS, 1% penicillin/streptomycin mixture, and 5% CMG14-12 conditioned medium. CMG14-12 conditioned medium was utilized as a source for macrophage colony-stimulating factor. Macrophages were washed thrice in Hank’s buffered saline solution (HBSS) and seeded at 2.0 × 10^5^ cells/mL in 12-well plates. Designated macrophages were activated using recombinant IFN-γ (5 ng/mL, Fujifilm Irvine Scientific) for 18–24 h. Antibiotics were omitted from macrophage culture medium 24 h prior to and throughout *Mtb* infection.

### Bacterial culture and macrophage infection

All experiments involving the use of *Mtb* were conducted inside a BSL-3 facility. *Mtb* H37Rv was used throughout this study. *Mtb* was grown in Middlebrook 7H9 medium containing 0.5% glycerol, 10% oleic acid-dextrose-catalase (OADC, BD Bioscience) supplement, and 0.02% tyloxapol at 37°C with mild shaking (100 rpm). For macrophage infection, mid-logarithmic *Mtb* was washed thrice in 1× phosphate buffered saline (PBS) containing 0.05% Tween 80 (TW80) and resuspended as a single-cell suspension in macrophage culture medium without antibiotics. Based on the OD_600_, the *Mtb* suspension was serially diluted to achieve the correct cell density necessary to obtain a multiplicity of infection (MOI) of 1 for macrophage infection assays. Single-cell suspension of the *Mtb* inoculum was achieved by allowing the prepared culture to incubate for 5 min at room temperature for any clumps to sediment. The top two-thirds of the *Mtb* suspension was used for macrophage infection. For infection, *Mtb* was added to macrophages at an MOI of 1. After 2 h of infection, extracellular bacteria were removed by washing macrophages thrice in HBSS (1.0 mL/well). Macrophage medium with or without pyrazinamide (200 and 400 µg/mL) was changed daily for the entire experiment. IFN-γ (5 ng/µL) was added every 2 days to designated cells. Infected macrophages were maintained in a 5% CO_2_ incubator at 37°C for 5 days. Macrophages were washed thrice in HBSS and lysed using 0.1% Triton-X solution at days 0, 1, 2, 4, and 5. Macrophage homogenates were serially diluted, plated on Middlebrook 7H10 agar supplemented with 0.5% glycerol and 10% OADC, and incubated at 37°C for 3 weeks until bacterial enumeration. Cell infection experiments were conducted in triplicate with two technical replicates for each treatment and timepoint.

### Mouse infection and drug treatment

Male and female C57BL/6J (*n* = 50), IFNGR1^−/−^ (*n* = 57), and MHC II^−/−^ (*n* = 50) mice were infected with ~100 CFU of *Mtb* using an inhalation exposure system (Glas-Col) as previously described (60). Nine to ten mice (*n* = 5 male and *n* = 4–5 female) from each strain were sacrificed by CO_2_ inhalation 24 h post-infection to determine the number of CFU established in the lungs. *Mtb* infection was allowed to progress for 3 weeks (pretreatment) to initiate the cell-mediated immune response, and 9–10 mice (*n* = 5 male and *n* = 4–5 female) were euthanized as before. Lungs, livers, and spleens were plated for bacterial enumeration. After the pretreatment, drug treatment occurred. PZA (150 mg/kg), INH (30 mg/kg), and sterile water (vehicle control) were administered to designated groups (*n* = 10–13 male and female per treatment and mouse strain) by oral gavage for 5 days a week for a total of 2 weeks. At the completion of drug treatment, mice were euthanized by CO_2_ inhalation, and organs were removed for bacterial enumeration.

### Enumeration of bacterial load

At the time of sacrifice, lungs, livers, and spleens were aseptically removed from the mice. All organs were mechanically disrupted in 1× PBS-TW80 using a tissue homogenizer. Organ homogenates were serially diluted and plated on MB7H10 agar supplemented with 0.5% glycerol, 10% OADC, and cycloheximide (10 mg/mL). Colonies were enumerated after at least 21 days of incubation at 37°C. Total bacterial burden was calculated based on organ volume.

### Statistical analysis

Comparisons of *in vivo* treatment groups at specified timepoints were analyzed using paired two-tailed *t*-tests, and *P* values were adjusted for multiple comparisons. Two-way ANOVA with Bonferroni correction was employed for BMDM infection assays. GraphPad Prism software v.10.2.2 was used to construct figures.

## SUPPLEMENTAL MATERIAL

Supplemental material aac.01264-25-s0001.docx Tables S1 and S2.

ASM does not own the copyrights to Supplemental Material that may be linked to, or accessed through, an article. The authors have granted ASM a non-exclusive, world-wide license to publish the Supplemental Material files. Please contact the corresponding author directly for reuse.
