# Supplementary material for: Essential role of MHC II in the antitubercular efficacy of pyrazinamide
Source: Antimicrob Agents Chemother. 2025 Dec 19;70(2):e01264-25. doi: 10.1128/aac.01264-25 (PMC12888911; doi:10.1128/aac.01264-25)
Supplement: Supplemental material — Tables S1 and S2. [file aac.01264-25-s0001.docx]

**Table S1: Log differences in statistically significant treatment comparisons in *Mtb* infected C57BL6/J mice BMDMs.**

|  |  | Day 4 |  |
| --- | --- | --- | --- |
| **C57BL6/J** | **Comparison** | **Log_10_ Difference** | ***P -*value** |
|  | PZA_400_ v. ND | 0.912 | 0.0278 |
|  | IFN-γ v. ND | 1.51 | 0.0201 |
|  | IFN-γ + PZA_200_ v. ND | 2.11 | 0.0184 |
|  | IFN-γ + PZA_400_ v. ND | 2.77 | 0.0180 |
|  | PZA_400_ v. PZA_200_ | 0.782 | 0.035 |
|  | IFN-γ v. PZA_200_ | 1.38 | 0.0223 |
|  | IFN-γ v. PZA_400_ | 0.6 | 0.05 |
|  | IFN-γ + PZA_200_ v. PZA_200_ | 1.977 | 0.0199 |
|  | IFN-γ + PZA_400_ v. PZA_200_ | 2.64 | 0.0013 |
|  | IFN-γ + PZA_200_ v. PZA_400_ | 1.19 | 0.0187 |
|  | IFN-γ + PZA_400_ v. PZA_400_ | 1.86 | 0.0192 |
|  | IFN-γ + PZA_400_ v. IFN-γ | 1.26 | 0.0189 |
|  | IFN-γ + PZA_400_ v. IFN-γ + PZA_200_ | 0.663 | 0.05 |
|  | **Day 5** | | |
|  | **Comparison** | **Log_10_ Difference** | ***P -*value** |
|  | PZA_400_ v. ND | 1.23 | 0.0079 |
|  | IFN-γ v. ND | 2.22 | 0.0041 |
|  | IFN-γ + PZA_200_ v. ND | 3.02 | 0.0038 |
|  | IFN-γ + PZA_400_ v. ND | 3.60 | 0.0031 |
|  | PZA_400_ v. PZA_200_ | 0.868 | 0.0345 |
|  | IFN-γ v. PZA_200_ | 1.85 | 0.0190 |
|  | IFN-γ v. PZA_400_ | 0.984 | 0.0269 |
|  | IFN-γ + PZA_200_ v. PZA_200_ | 2.65 | 0.0178 |
|  | IFN-γ + PZA_400_ v. PZA_200_ | 3.24 | 0.0034 |
|  | IFN-γ + PZA_200_ v. PZA_400_ | 1.78 | 0.0188 |
|  | IFN-γ + PZA_400_ v. PZA_400_ | 2.37 | 0.0045 |
|  | IFN-γ + PZA_200_ v. IFN-γ | 0.8 | 0.0322 |
|  | IFN-γ + PZA_400_ v. IFN-γ | 1.38 | 0.0082 |
|  | IFN-γ + PZA_400_ v. IFN-γ+ PZA_200_ | 0.585 | 0.0356 |

**Table S2: Log differences in statistically significant treatment comparisons in *Mtb* infected C57BL6/NJ mice BMDMs.**

|  |  | Day 4 |  |
| --- | --- | --- | --- |
| **C57BL6/NJ** | **Comparison** | **Log_10_ Difference** | ***P -*value** |
|  | PZA_400_ v. ND | 0.692 | 0.0394 |
|  | IFN-γ v. ND | 1.04 | 0.0244 |
|  | IFN-γ + PZA_200_ v. ND | 1.33 | 0.0201 |
|  | IFN-γ + PZA_400_ v. ND | 1.85 | 0.0174 |
|  | PZA_400_ v. PZA_200_ | 0.360 | 0.0031 |
|  | IFN-γ v. PZA_200_ | 0.709 | 0.0003 |
|  | IFN-γ + PZA_200_ v. PZA_200_ | 1 | 0.0003 |
|  | IFN-γ + PZA_400_ v. PZA_200_ | 1.54 | 0.0002 |
|  | IFN-γ + PZA_200_ v. PZA_400_ | 0.64 | 0.0461 |
|  | IFN-γ + PZA_400_ v. PZA_400_ | 1.18 | 0.0211 |
|  | IFN-γ + PZA_400_ v. IFN-γ | 0.831 | 0.0032 |
|  | IFN-γ + PZA_400_ v. IFN-γ + PZA_200_ | 0.54 | 0.0010 |
|  | **Day 5** | | |
|  | **Comparison** | **Log_10_ Difference** | ***P -*value** |
|  | PZA_400_ v. ND | 0.953 | 0.0427 |
|  | IFN-γ v. ND | 1.34 | 0.0322 |
|  | IFN-γ + PZA_200_ v. ND | 1.87 | 0.0282 |
|  | IFN-γ + PZA_400_ v. ND | 2.77 | 0.0269 |
|  | PZA_400_ v. PZA_200_ | 0.6 | 0.0077 |
|  | IFN-γ v. PZA_200_ | 0.985 | 0.0034 |
|  | IFN-γ v. PZA_400_ | 0.389 | 0.0003 |
|  | IFN-γ + PZA_200_ v. PZA_200_ | 1.51 | 0.0025 |
|  | IFN-γ + PZA_400_ v. PZA_200_ | 2.41 | 0.0022 |
|  | IFN-γ + PZA_200_ v. PZA_400_ | 0.914 | <0.0001 |
|  | IFN-γ + PZA_400_ v. PZA_400_ | 1.82 | <0.0001 |
|  | IFN-γ + PZA_200_ v. IFN-γ | 0.524 | 0.0090 |
|  | IFN-γ + PZA_400_ v. IFN-γ | 1.43 | 0.0033 |
